# Storm Response of Fluvial Sedimentary Microplastics

**DOI:** 10.1038/s41598-020-58765-2

**Published:** 2020-02-05

**Authors:** Annie Ockelford, Andy Cundy, James E. Ebdon

**Affiliations:** 1 Centre for Aquatic Environments, University of Brighton, Brighton, BN2 4GJ UK; 2 School of Ocean and Earth Science, National Oceanography Centre (Southampton), University of Southampton, Southampton, SO14 3ZH UK

**Keywords:** Hydrology, Environmental impact, Climate-change impacts

## Abstract

Up to 80% of the plastics in the oceans are believed to have been transferred from river networks. Microplastic contamination of river sediments has been found to be pervasive at the global scale and responsive to periods of flooding. However, the physical controls governing the storage, remobilization and pathways of transfer in fluvial sediments are unknown. This means it is not currently possible to determine the risks posed by microplastics retained within the world’s river systems. This problem will be further exacerbated in the future given projected changes to global flood risk and an increased likelihood of fluvial flooding. Using controlled flume experiments we show that the evolution of the sediment bed surface and the flood wave characteristics controls the transition from rivers being ‘sinks’ to ‘sources’ of microplastics under flood conditions. By linking bed surface evolution with microplastic transport characteristics we show that similarities exist between granular transport phenomena and the behavior, and hence predictability, of microplastic entrainment during floods. Our findings are significant as they suggest that microplastic release from sediment beds can be managed by altering the timing and magnitude of releases in flow managed systems. As such it may be possible to remediate or remove legacy microplastics in future.

## Introduction

The microplastic burden in aquatic environments is now recognised as a potential threat to human and environmental health^1,2^. To date, the focus has been on marine environments where there are an estimated 4.85 trillion microplastic (<5 mm) particles in the global ocean^3^. However, up to 80% of the plastics in the oceans are believed to have been transferred from river networks^4–6^. Whist recent work indicates microplastic contamination of river sediments is pervasive at the global scale^7^ there remains a paucity of research on the pathways and mechanisms of microplastic transfer, particularly between coarse and mixed bed granular systems and the oceans.

As well as acting as a source, data from the sedimentological literature points towards river beds as being resilient sinks for contaminants including heavy metals, organic contaminants and microorganisms such as viral pathogens^8–10^. Limited research has shown this to also be the case for microplastics, where they can accumulate in sediments at an order of magnitude higher than in the overlying water column^11^. In some catchments there are greater than 500,000 microplastic particles per m^−2^ of river bed^7^. Further, a limited number of studies have suggested that the coarser and more heterogeneous the granular bed material the higher the retention of microplastics^12,13^. This suggests that coarse granular rivers have the potential to be key global drivers in microplastic dynamics yet remain relatively overlooked.

Whether the river bed acts as a source or sink of microplastic is controlled by the properties of the sediment bed and the interactions between the sediment dynamics and hydraulics at the fluid-sediment interface^6^. Erosion and deposition of the sediment bed surface will control the extent to which microplastics either become incorporated into and buried, or eroded from, the bed. The vertical depth of the sediment bed across which exchanges can occur is known as the active layer^14–16^. During flood events the active layer can be completely mobilised^17,18^. Consequently, active layer dynamics will drive the vertical exchange of microplastics such that its remobilisation has the potential to generate large scale release of microplastics from within the bed. Limited field evidence supports this, with threefold increases in microplastic concentration observed in surface water of the Cooum River, India^19^ during a 2015 flood event and a 64% reduction of microplastic concentrations observed in river sediments from the River Irwell, UK following a 2015/16 flood^7^ event. Data also showed there to a significant heterogeneity of types of plastics found with beads, fibres and fragments dominating samples. However, the reduction in concentration of plastics, irrespective of the type or density was specifically linked to sediment bed erosion processes. In terms of the non-buoyant particles, this implies that there is a flow threshold above which rivers may move from being a sink to a source of microplastic. If this is the case it means that fluvial processes have the capacity to significantly increase the microplastic flux into our oceans under flood conditions. This is particularly salient in light of the predicted increases to global flood risk with suggestions that most major fluvial systems are likely to experience a greater number of higher magnitude, more frequent, flood events^20–23^.

In this paper, we report a series of novel, mobile-bed laboratory flume experiments designed to explicitly quantify the effect of flood wave characteristics on the relationship between river sediment bed surface development and microplastic flux characteristics. In doing so, we highlight that there are thresholds at which the sediment bed transitions from being a sink to a source of microplastics and back again at different points during the flood wave. We also show that microplastic flux is dependent on the flood wave characteristics which have implications for the prediction of microplastic dynamics and the management of water and biological resources, in rivers which are characterised by different flow regime characteristics.

## Results

### Bed surface dynamics controlling microplastic flux

**Figure 1:**
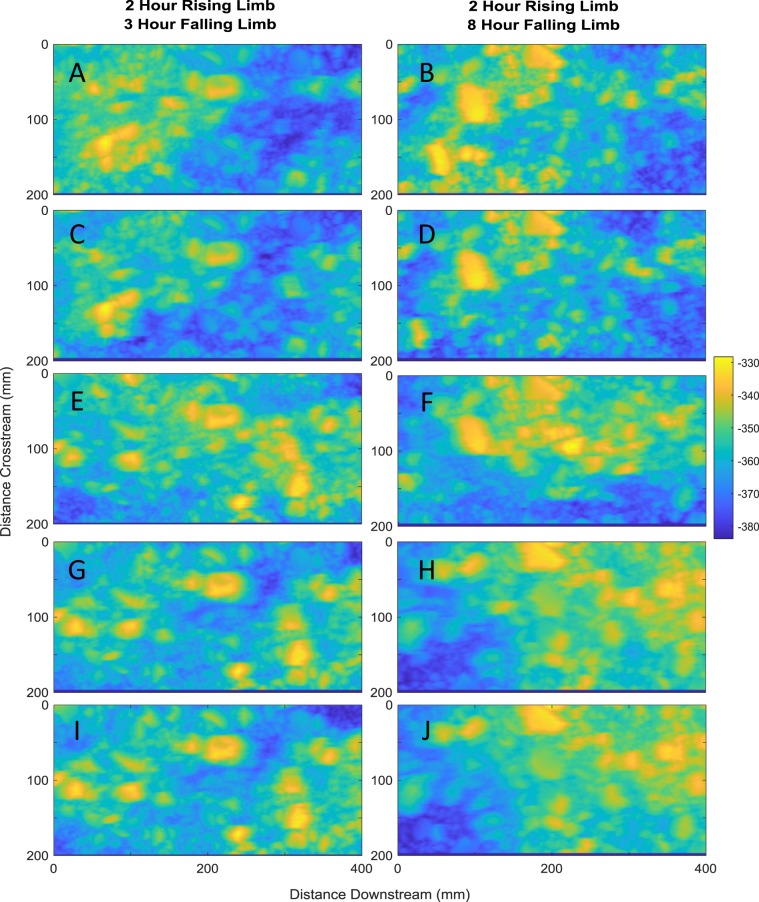
Digital Elevation Models (DEMs) of the bed surface topography for different phases through the flood wave. The left hand column refers to the 2 hour rising limb and 3 hour falling limb experiment whilst the right hand column refers to the 2 hour rising limb and 8 hour falling limb experiment. Sequentially from the top the DEMs plot the unarmoured starting surface (**A**,**B**), the surface at τ*/τ*c = 1.06 on the rising limb (**C**,**D**), the surface at the peak discharge (**E**,**F**), the surface at τ*/τ*c = 1.06 (**G**,**H**) on the falling limb and the final surface at the end of the flood wave (**I**,**J**). Units are given in mm below an arbitrary datum, in this case the surface of the laser scanner.

The depth of the active layer is typically defined as being equivalent to that of the maximum grain (D_max_) size^24,25^ and so the evolution of active layer depth herein (Table 1) is scaled relative to this maximum grain size. Evolution of the surface through the flood wave influences the surface grain size distribution and arrangement and may serve to mediate active layer thickness and hence exchange depths. By quantifying bed surface evolution through the flood wave under controlled flume conditions we show that there are discrete periods of bed aggradation and degradation (Fig. 1), occurring at two different scales; macro bed-scale and meso grain-scale. Further, surface evolution and active layer thickness during the flood wave are seen to have distinct thresholds on the rising and falling limbs which appear to be related to the stability of the median grain size.

**Table 1:** Armour layer depth derived from the laser scan data as detailed in the methodology as a function of τ*/τ*_c_.

τ*/τ*_c_ value	Armour Layer Depth 2 Hour Rising Limb, 3 Hour Falling Limb	Armour Layer Depth 2 Hour Rising Limb,8 Hour Falling Limb
**0.81**	**0.82**	**0.82**
**0.90**	**0.82**	**0.86**
**0.99**	**0.85**	**0.86**
**1.08**	**0.87**	**0.91**
**1.17**	**0.89**	**0.94**
**1.26**	**0.90**	**0.95**
**1.35**	**0.95**	**0.97**
1.26	0.97	1.30
1.17	0.97	1.36
1.08	0.97	1.29
0.99	0.97	1.28
0.90	0.94	1.24
0.81	0.85	1.24

Numbers in bold denote the rising limb values.

**Figure 2:**
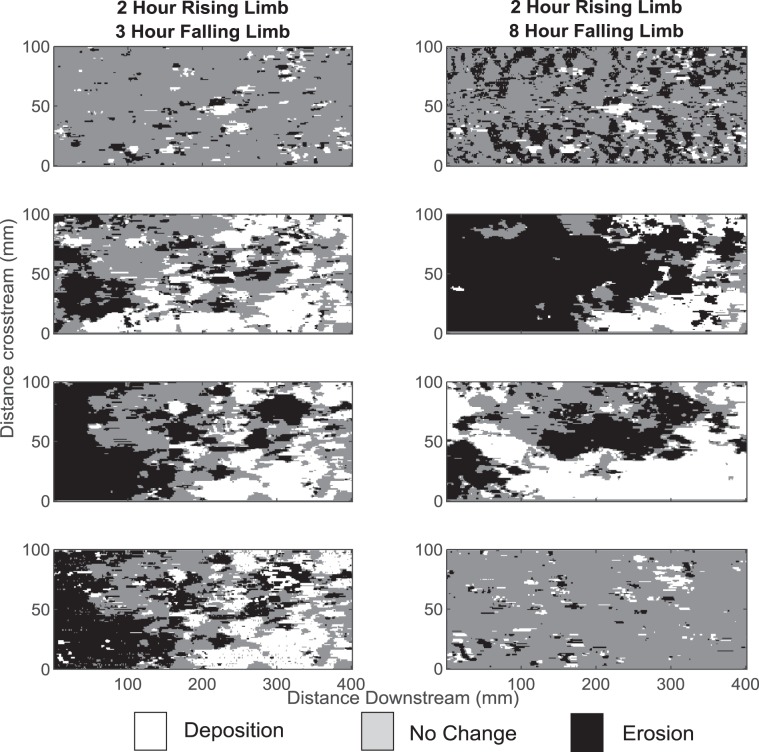
Digital Elevation Models of Difference (DoD). On each image white represents deposition, grey represents no change and black represents erosion. Image pair A plot the elevation change between 1st and 2nd step of the rising limb (between τ*/τ*c values of 0.81 to 0.9), Image pair B plot the elevation change between the final step of the rising limb and the peak discharge (between τ*/τ*c values of 1.26–1.35), Image pair C plot the elevation change between the peak discharge and the first step of the falling limb (between τ*/τ*c values 1.35 and of 1.26) and Image pair D plot the elevation change between the penultimate and final step of the falling limb (between τ*/τ*c values of 0.9 to 0.81).

In order to show applicability to gravel bed rivers beyond those reported in this paper, results are discussed in terms of excess critical shear stress of the median grain size (τ*/τ*_c_ where τ* is the dimensionless shear stress and τ*_c_ is the critical dimensionless shear stress for the median grain size (D_50_) and the ratio represents the excess critical shear stress)). During the early parts of the flood wave (τ*/τ*_c_ values up to 0.9) the median grain size is stable on the bed surface and there is no net aggradation or degradation of the bed surface (Fig. 2).

Instead, the surface is seen to evolve at the bed-scale by the gravity-driven process of kinematic sorting, in which finer fractions of the bed surface material infiltrate into the bed^26,27^. This leads to the coarsening of the surface and the development of an armour layer, as evidenced by an 8% and 11% coarsening in the D_50_ and D_84_, respectively^28,29^. Grain scale processes act alongside the bed scale evolution such as to develop distinct structures which serve to stabilise localised areas of the bed surface (Fig. 1). Under these conditions the sediment bed will act as a sink for microplastics since the coarsening of the surface limits vertical exchanges across the active layer and the bed surface remains relatively stable.

As discharge increases through the flood wave and τ*/τ*_c_ increases from 0.9 to 0.98, active layer depth increases from 0.82D_max_ to 0.87D_max_ (Table 1). The median grain size fraction becomes mobile at τ*/τ*_c_ ranges of 1.06 to 1.15 and armouring continues such that the D_50_ and D_84_ coarsen by a further 7.6% and 2.5%. However, increasing proportions of the bed surface also become active as the bed moves from stability to instability. Active layer depths under these flow conditions continue to increase to 0.90D_max_. Under peak discharge active layer depth increases to 0.95D_max_ which happens alongside the breakup of the entire armour layer. This means that sediment can be exchanged from deeper within the bed and under these conditions the sediment bed will switch from being a sink to a source of microplastics.

The falling limb shows more complex dynamics. During the shorter falling limb as the τ*/τ*_c_ value decreases from 1.26 at the peak discharge to 0.99, active layer depth remains stable but is slightly deeper (2%) than that experienced on the rising limb. As τ*/τ*_c_ falls back to base flow conditions, armour layer depth decreases to pre-flood depths and the bed once again reverts back to being a potential sink for microplastics. Conversely, for the longer duration falling limb although active layer depth decreases as τ*/τ*_c_ falls values are up to 40% greater than both that observed on the rising limb and those observed during the shorter falling limb experiment. This indicates that under these flood conditions microplastics can continue to be mined from deeper within the bed and hence act as a source of microplastics even as the flood wanes. Further, active layer depths under these conditions never return to pre-flood depths which suggests that the switch back to sink conditions is fundamentally linked to the hydrograph characteristics.

### Flood driven microplastic flux

**Figure 3:**
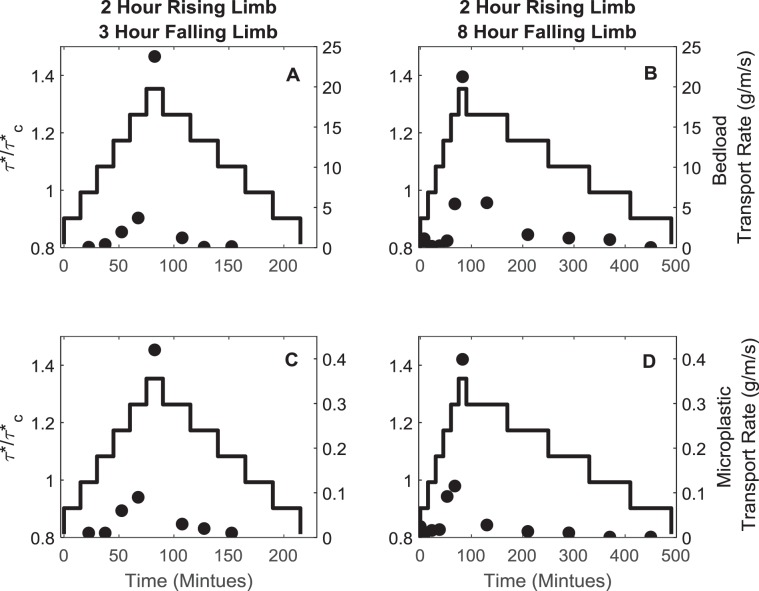
Bedload (panels A and B) and microplastic (panels C and D) flux characteristics as a function of time through the flood wave depicted as solid black symbol. Excess critical shear stress (τ*/τ*_c_) plotted as a function of time is given by the solid black line. Bedload and microplastic flux can be described by an exponential decline on the falling limb with a rate constant of 0.012 and 0.014 for the bed and microplastic load respectively for the 2 hour rising limb and 3 hour falling limb experiment and a rate constant of 0.037 and 0.034 for the bed and microplastic load respectively for the 2 hour rising limb and 8 hour falling limb experiment.

In the reported experiments microplastic flux will be controlled by the characteristics of the plastic itself and the changes to be bed surface. The plastics used in the experiments (recycled rigid polyvinyl chloride pellets, D_50_ of 3.8 mm, density of 1.33 g/cm^3^) were observed to behave in a similar manner to that of the sediment in terms of hop lengths, behaviour within the bed and interaction with the non-plastic particles. Changes to the active layer dynamics and the concomitant surface evolution links to periods of suppressed and elevated microplastic flux (Fig. 3). An increase in both bedload and microplastic load is observed as discharge increases through the rising limb and, akin to bed surface development, can be defined by three thresholds. The first of these happens as τ*/τ*_c_ values approach 0.9 where the bed surface is undergoing armouring which serves to stabilise the bed surface and does not permit microplastic transport. As τ*/τ*_c_ values increase to values of between 0.9 and 0.98 individual grain-scale structures on the bed surface break down in response to increasing shear stress, leading to increases of up to 17% and 39% in the bed and microplastic load respectively. The final threshold happens at the peak flood discharge during mobilisation of the armour layer where up to 78% and 53% of the total bed and microplastic load is entrained respectively. This equates to a factor of 6 increase in microplastic flux between the point preceding the peak discharge and the peak discharge, and thus it is at this point that the sediment bed is evidenced to have transitioned from being a microplastic sink to a microplastic source.

The time taken for the bed to return to stability, as defined by a reduction of microplastic flux to pre-flood magnitudes, and resume its role as a sink depends on the shape of the flood hydrograph. While, the microplastic load decreases exponentially irrespective of the falling limb form, both microplastic flux and the rate of reduction are responsive to the duration of the falling limb; the longer the duration of the falling limb, the higher the microplastic flux but the quicker the rate of reduction (Fig. 3). Further, during both falling limbs the bedload transport rate reduces more quickly than the microplastic flux (Fig. 3) and the D_50_ of the bedload fines. Bedload fining is inversely proportional to bed surface coarsening, suggesting that the bed is re-armouring by stabilisation of increasingly coarse clasts which controls the rate of reduction in the microplastic flux.

**Figure 4:**
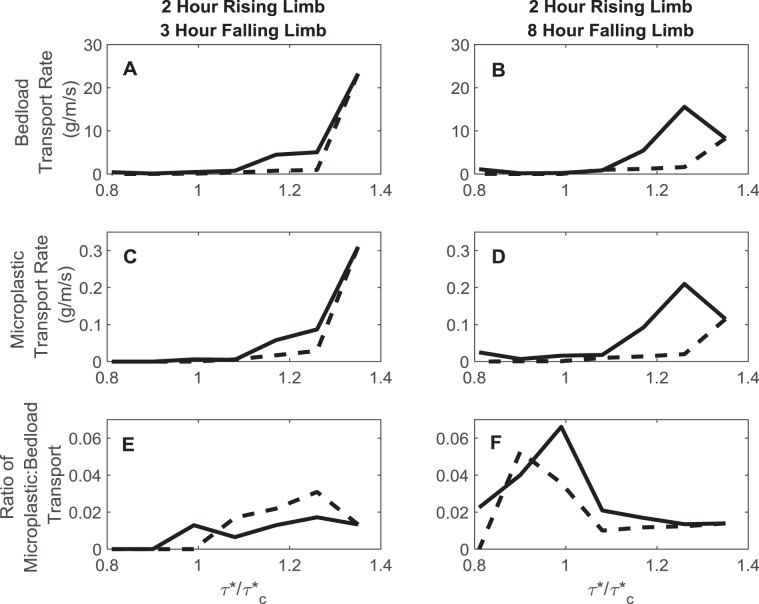
Hysteresis plots of bedload transport rate (**A**,**B**) microplastic transport rate (**C**,**D**) as a function of excess critical shear stress (τ*/τ*c). Clockwise hysteresis is evidenced where bedload and microplastic fluxes are higher on the rising limb (solid black line) compared to the falling limb (dashed black line). The ratio of microplastic flux to bedload flux is also plotted as a function of excess critical shear stress (**E**,**F**).

Both the bedload and the microplastic load show a clockwise hysteretic response (Fig. 4). However, despite the fact that the duration of the falling limb is longer than that of the rising limb in both experimental cases, it is the rising limb which drives the microplastic flux accounting for up to 97% of the flux over the entire flood event. The strength and timing of this hysteretic response in both microplastic and bedload flux is controlled by the shape of the flood wave where short flood waves only develop hysteresis in the bedload flux. Comparatively, for the longer hydrographs both the bedload and microplastic load show significant hysteresis which persists for all shear stresses. However, the transport of microplastics relative to the bedload shows that the microplastics are transported in greater relative quantities during the falling limb of shorter hydrographs.

## Discussion

According to Hurley *et al*.^7^ further work to identify the quantities and mechanisms of microplastic transfer from river catchments to oceanic waters is fundamental to better understand, and by inference mitigate, the global distribution of microplastic contamination. These authors note a reduction in microplastic concentration within the sediment bed following floods and suggest that fluvial processes therefore have the capacity to rapidly cleanse river channel beds of microplastic contamination. However, our findings suggest that the situation is significantly more complex than previously thought, as there are thresholds at which the sediment bed transitions from being a sink to a source of microplastics and back again at different points during the flood wave. These thresholds occur in response to changes in the relationship between the granular bed mechanics and the discharge characteristics, with microplastic flux controlled by active layer dynamics.

Under low-flow conditions the bed develops an armour layer which serves to stabilise the bed surface and thus limits microplastic “winnowing” or release, such that the sediment bed acts as a sink^30^. As discharge increases through the flood wave small-scale bed instabilities and increasing active layer depths promote the vertical exchange of microplastics^31,32^. The peak discharge is a key parameter in the control of microplastic flux, accounting for up to 53% of the total load transported during the flood. We have shown that bulk release of microplastics is controlled by the wholesale breakdown of the armour layer which serves to increase active layer depth and promote microplastic transport. As discharge decreases during the falling limb the bed re-stabilises via surface coarsening and reduction of active layer depth. However, active layer depth is strongly influenced by the hydrograph shape whereby, the longer the falling limb the deeper the active layer depth^33^. This has implications for the likely persistence of microplastic release even beyond the peak discharge. The fact that these thresholds appear to be particularly sensitive to the stability of the median-sized grains (i.e. D_50_) is significant for the transferability of these results as this grain size is typically used to predict critical entrainment thresholds which underpin many sediment transport formulae^34,35^. This implies that microplastic transport beyond the experimental conditions reported herein can be modelled using standard sediment transport equations. However, whilst these patterns hold true for the microplastic pellets used in these experiments it is recognised that contamination in real rivers is often far more complex and characterised by microplastics which have a range of sizes, densities and morphologies^7,36–39^. Given these characteristics will affect microplastic propensity to be eroded from, or retained within, the sediment bed as well as their entrainment characteristics once they are eroded^40^ future research should be directed towards understanding the implications of this for modelling their fate.

Our data have shown that the hydrograph characteristics are a fundamental control on microplastic flux in coarse granular rivers. This has implications for the prediction of microplastic dynamics, and also the management of water and biological resources, in rivers which are characterised by different flow regime characteristics. These include sub-tropical and temperate rivers which are major suppliers of oceanic microplastics^42^. Given the predicted increases to the frequency and magnitude of fluvial flood events^20–23^, our results also have significant implications for the management of global river systems, and microplastics, in light of climate change. A change in the frequency of flood events means that the periods of low, inter-flood flows will decrease. Given that our data has shown that periods of low shear stress serve to consolidate the bed (enhancing its capacity to act as a sink) any increase in flood frequency is likely to increase microplastic exchanges between the sediment bed and the overlying water column. Further, the transition between the bed acting as a sink and the bed acting as a source of microplastics is likely to occur earlier in the flood than previously assumed. A change in the magnitude of flood events is likely to progressively degrade bed stability and shift the balance between sink and source which has implications for both the release of legacy microplastics, which are already buried as well as the flux characteristics of new loadings into the channel. Finally, increased pressure on water resources means more sophisticated environmental flow guidelines are needed in order to ensure and maintain ecosystem health and enable effective water resource management planning. For flow-managed systems, our data have shown that by altering the timing and magnitude of flow releases there is the potential to promote the sink behaviour of sediment beds. This is significant, as our findings suggest that it may be possible to limit subsequent microplastic release into riverine and marine waters within such systems.

## Materials and Methods

Flume experiments were conducted to quantify the effects of flood-wave (hydrograph) shape on microplastic flux in gravel bed rivers. The characteristics of the hydrographs were not directly scaled to natural river hydrographs, however a review of the hydrograph characteristics used previously within the literature, together with an analysis of 50 floods in UK gravel bed river catchments (for a catchment size of ~100 km^2^) was undertaken. Specifically, the ratio of the rising to falling limb duration was analysed with 95% of the ratios falling between 1.5 and 4. Therefore two different hydrographs were analysed in this study with a ratio of the rising to falling limb duration of 1.5 and 4.

Experiments were performed within a glass sided, flow recirculating flume of rectangular cross section (8.2 m × 0.6 m × 0.5 m). To prevent scour and to induce turbulent boundary conditions, 2.1 m of immobile sediment was placed directly downstream of the water inlet and no measurements were made in the final 1.7 m of the flume to avoid draw-down effects. This gave an effective working length of 4.4 m, which was covered with mobile test sediments to a depth of 0.1 m. Natural river sediments ranging between 1 and 45 mm were sieved into half *phi* fractions, painted by grain fraction to allow for identification, and recombined to form a grain size distribution with an inclusive graphic sorting coefficient^43^ of 1.5 and D_50_ of 9 mm. Recycled rigid polyvinyl chloride (PVC) microplastic pellets (nurdles) ranging from 3–5 mm (D_50_ (median nurdle size) of 3.8 mm with a density of 1.33 g/cm^3^)) were used within the experiments. The pellets were not treated in any way prior to being added into the flume where they were mixed into the bulk sediment mixture at the start of the experiment at a 1% concentration by weight. In all cases, flow was uniform through the test section, with no measured acceleration or deceleration along the length of the flume and a relatively stable water surface.

## Experimental Procedure

During each experiment the flume was set to the desired slope (0.009 m/m) and the bed screeded to match the flume slope. Three experimental phases were then run; (a) an initial bedding-in period; (b) an armouring flow period and; (c) a flood wave. During the bedding-in period the sediment bed was water-worked for 30 minutes at a dimensionless shear stress of 0.0026 to remove any unstable grains and displace any air pockets. Measurements of the bed surface were then made by stopping the pump and allowing the bed to drain naturally. This was observed to stop transport immediately without causing any obvious disturbance to the bed surface, e.g. by drawing fines down through the bed surface and thus ensured minimal surface disruption^30,41,44,45^. For all experiments a test section of 200 mm by 400 mm was then scanned using a point laser mounted on an x, y traverse 3 m from the flume inlet. This collected a point cloud of elevation data which was post processed to produce a Digital Elevation Model (DEM) of each bed surface at 1 mm resolution. Active layer depth was calculated as being the range of elevations from the highest roughness crest down to the lowest layer tending to zero porosity; this is commonly assumed to correspond to the active layer depth when laser-based measurement techniques are employed^25,46^. Finally, whilst the pump was stopped a photograph was taken of the bed surface. The surface grain-size distribution was sampled by superimposing a grid over the photographs and noting the colours of grains falling under the grid intersections^47^. Each sample comprised 200 point counts taken from the same 200 by 400 mm area of the test section using a 20 mm square grid and provided a statistically tractable estimate of grain size distribution. Since the grains were coloured it also avoided bias related to partial burial and grain orientation^48^.

**Figure 5:**
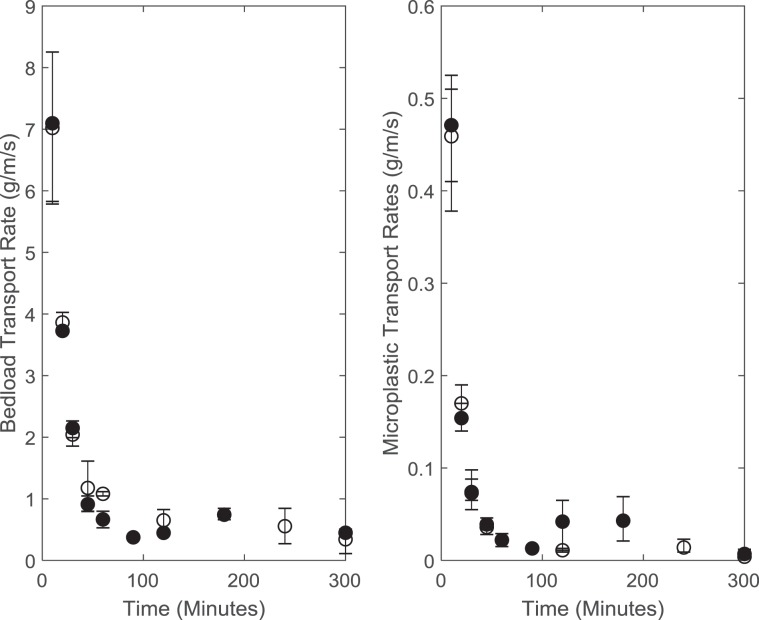
Bedload transport rate (left hand figure) and microplastic transport rate (right hand figure) through time in the armouring flow period. On both figures data points represent the average transport rate taken from the three runs and error bars represent the maximum and minimum recorded transport rate for each time period. Closed circles represent data collected during 2 hour rising limb and 3 hour falling limb experiments and open circles represent data collected during the 2 hour rising limb and 8 hour falling limb experiments.

To ensure the bed surface was representative of that found in the field, the flow was carefully restarted and the second experiment phase, the armouring flow period, was run using a dimensionless shears stress of 0.044, which generated conditions representative of partial mobility. The duration of the armouring flow was chosen in line with pilot studies, which established that fluctuations in transport rate decreased markedly over the first 5 hours of each experimental run and had fallen to ~1% of the initial fluctuations. This ensured that the armoured surfaces generated in each experiment were produced by a sustained period of quasi-steady transport^30,31^. During the armouring period flow depth was set at 120 mm to maintain a width depth ratio of 5 and an initial relative depth ratio of 6.3 and 13.3 for the D_84_ and D_50_ respectively. These width depth ratios are high enough for two dimensional flow to be generated^49,50^ and are representative of gravel bed rivers measured in the field. Any sediment or microplastics which were transported during the armouring flow period bedload were collected in a sediment trap at the downstream end of the working section and removed. Although the temporal change in microplastic and bedload flux during the armouring period is not reported in the main paper, the pattern of reduction in both is representative of previous armouring experiments^30^ (Fig. 5). At the end of the armouring flow the pump was once again stopped and the bed surface scanned and surface photographs taken. This surface represented the armoured start surface and is the equivalent of a sediment bed which has been water-worked by inter-flood flows in the field.

Flow during the final experimental phase was characterized by a period of unsteady flow simulating a rising and falling limb of a flood wave. During this period flow was increased incrementally in dimensionless shear stress values of 0.0026 steps from a base flow until a maximum shear stress was reached. Dimensionless shear stress ranged from 0.033 to 0.059 during the hydrograph. The duration of the flow steps in the rising and falling limb varied in length with each step being 17 minutes for the rising limb and either 30 minutes or 80 minutes for the falling limb. Sediment was collected for each step of the hydrograph, removed and sieved into the original size fractions. Any microplastics which were entrained were collected in the bedload trap at the same sampling frequency as the bedload data and manually removed to be weighed. Each experiment was repeated three times. For two of the three runs the flume was not stopped and only bedload and microplastic flux measurements were taken with no scans. On the third run the flume was stopped every step of the hydrograph as described above in order to take scans. This approach was adopted to assess the impact that stopping the flume had on the transport dynamics. Data shows that bedload transport rates are only up to 4% different between runs where the flume was and was not stopped. However, rather than averaging the results the reported bedload and microplastic flux rates (Figs. 3 and 4) are those associated with the experiment where the flume was stopped and scans taken such that the links between the different bed topographies could be explicitly linked with the associated bedload and microplastic fluxes.

## Data Availability

All datasets are available from the corresponding authors upon request.
